# Microwave Treatment vs. Conventional Pasteurization: The Effect on Phytochemical and Microbiological Quality for Citrus–Maqui Beverages

**DOI:** 10.3390/foods13010101

**Published:** 2023-12-27

**Authors:** Francisco J. Salar, Alejandro Díaz-Morcillo, José Fayos-Fernández, Juan Monzó-Cabrera, Paola Sánchez-Bravo, Raúl Domínguez-Perles, Pablo S. Fernández, Cristina García-Viguera, Paula M. Periago

**Affiliations:** 1 Laboratorio de Fitoquímica y Alimentos Saludables (LabFAS), Department Food Science and Technology, CSIC, CEBAS, Campus Universitario-25, Espinardo, 30100 Murcia, Spain; fjsalar@cebas.csic.es (F.J.S.); psanchez@cebas.csic.es (P.S.-B.); rdperles@cebas.csic.es (R.D.-P.); 2 Departamento de Tecnologías de la Información y las Comunicaciones, Universidad Politécnica de Cartagena (UPCT), 30202 Cartagena, Spain; alejandro.diaz@upct.es (A.D.-M.); jose.fayos@upct.es (J.F.-F.); juan.monzo@upct.es (J.M.-C.); 3 Centro de Investigación e Innovación Agroalimentaria y Agroambiental (CIAGRO), Universidad Miguel Hernández de Elche (UMH), Carretera de Beniel km 3.2, 03312 Orihuela, Spain; 4 Unidad Asociada de Calidad y Evaluación de Riesgos de Alimentos, CEBAS (CSIC)-UPCT, 30100 Murcia, Spain; pablo.fernandez@upct.es (P.S.F.); paula.periago@upct.es (P.M.P.); 5 Agronomic Engineering Department, Universidad Politécnica de Cartagena (UPCT), Paseo Alfonso XIII, 48, 30203 Cartagena, Spain

**Keywords:** sweetener, thermal treatment, functional quality, flavanones, anthocyanins, vitamin C

## Abstract

This study uncovered the impacts of microwave (MW) treatments compared to conventional pasteurization (TP) on the quality of functional citrus–maqui beverages, with added sucrose or stevia. The influence of these thermal treatments on the microbiological burden and phytochemical composition was determined by processing under two MW power levels (600 W and 800 W) and TP at 85 °C for 15 s for 60 days at room temperature (20 °C). The results indicated that, beyond the microbiological quality achieved in the juices treated by both MW and TP technology, there were no differences among the treatments regarding the stability of vitamin C, anthocyanin, and flavanone concentrations. However, anthocyanins were more stable in those beverages with sucrose added, rendering a better red color. Besides, all treatments ensured microbiological stability throughout the entire storage time. In conclusion, MW treatment could be considered as an alternative to TP, which ensures microbial safety, protecting functional compounds associated with health effects.

## 1. Introduction

Nowadays, consumers have a growing interest in the consumption of functional foods (due to the increasing interest in healthy lifestyles), minimally processed and with a long shelf life, such as drinks, with a high content of bioactive compounds responsible for healthy effects [1]. In this sense, the intake of plant-based foods, rich in phytochemicals, is related to inflammation- and oxidative-stress-preventing capacities, as well as the reduction in some types of cancer and other degenerative pathophysiological conditions [2,3]. Moreover, in recent decades, the consumption of sugar-sweetened drinks has been attributed to an enhanced risk of an array of pathologies, namely diabetes mellitus, obesity, and cardiovascular diseases, among others [4]. Consequently, the agro-food industries are pursuing the design and development of new products with a low sugar content, which can lead to preserving the key hypoglycemic food compounds of beverages, by adding non-caloric sweeteners, such as stevia [5].

In this frame, recently, our group has developed new beverages based on maqui berries (*Aristotelia chilensis* (Mol.) Stunz) and citrus fruits. The use of different sweeteners in the development of these beverages has allowed setting up alternatives to sugar, highlighting their influence on bioactive compounds’ stability in conventional heat pasteurization (TP) and high-hydrostatic-pressure processing [6,7].

Nevertheless, the processing of beverages using conventional pasteurization presents some disadvantages [7], even though this technology ensures stability during processing and shelf life, as its influence on certain bioactive compounds has to be carefully studied for each new beverage. For this reason, the application of new technologies should be explored, such as microwave treatment (MW), a possible thermal alternative that does not affect the content of phytochemicals present in the raw material [8]. Microwave heating and conventional (convective) heating have different physical mechanisms. Convective heating is produced by the conduction of heat from the surface throughout the processed material. Microwave heating is a combination of both direct heat generation inside the material and the conductive heating process. Microwave heat generation is produced by the agitation of polar molecules, which try to follow the electric field variation.

Currently, the use of MW treatment in food processing, especially in liquid foods, has increased, mainly due to its high capacity to reduce heating time. Moreover, it is a precise process, with the advantages of homogeneity, easy maintenance, and efficient space utilization [9,10]. Microwaves are a form of electromagnetic radiation in which energy is converted to heat. MW radiation ranges from 300 MHz to 300 GHz, although only very narrow bands are permitted for industrial, scientific, and medical (ISM) applications, mainly around 915 MHz and 2.45 GHz. Exposure times vary from 10 to 300 s, and temperatures from 45 °C up to 91 °C [11,12]. Thanks to the shorter exposure time to high temperatures and the energy supplied, MW treatment could also help to preserve phytochemical compounds and sensory properties [13,14]. However, the processing conditions associated with this treatment have to be optimized; therefore, microwave treatment could still affect the flavor and nutritional values of the juice, although to a lesser extent than TP [9].

Given these antecedents, the present work uncovers the advantages of applying MW heat treatments by considering different power levels (600–800 W, selected according to previous descriptions on the MW power requirements for ensuring microbial safety in beverages [15,16]) for maqui and citrus-based beverages, with different sweeteners added, compared to the TP process (85 °C for 15 s), to minimize the loss of relevant bioactive compounds (flavonoids and vitamin C), thus ensuring the biological safety of the final product.

## 2. Materials and Methods

### 2.1. Chemicals and Reagents

Cyanidin 3-O-glucoside was obtained from TransMIT (Geiben, Germany) and Hesperidin from Sigma-Aldrich (St. Louis, MO, USA). Brilliant Green Bile 2% Broth (BGBB), Plate Count Agar (PCA), buffered peptone water, Man Rogosa Sharpe Agar (MRSA), and Rose Bengal Agar (RBA) were acquired from Scharlab (Barcelona, Spain). Methanol, acetonitrile, formic acid, and ethylenediaminetetraacetic acid disodium salt 2-hydrate (EDTA), from Panreac (Barcelona, Spain). Dehydroascorbic and L-ascorbic acids (DHAA and AA, correspondingly) were purchased from Sigma-Aldrich (St. Louis, MO, USA) and Acros Oganics (Thermo Fisher Scientific Inc., Madrid, Spain), respectively. All solutions were prepared with ultrapure water from a Milli-Q Advantage A10 ultrapure water purification system (Millipore, Burlington, MA, USA).

### 2.2. Ingredients

Fresh, dry, organic maqui powder, citrus juices, and the sweeteners (stevia and sucrose) were provided as previously described by Salar et al. [6].

### 2.3. Experimental Design

#### 2.3.1. Beverage Preparation

The citrus–maqui beverage formulation was carried out according to previous studies of the research group [6]. In summary, the base beverage was obtained by mixing maqui powder with citrus juices to obtain the base drink. Then, the sweetener was added and homogenized to obtain the finished beverage, resulting in a homogenous solution with suspended particles originating from the fruits used in the formulation.

#### 2.3.2. Microwave Processing

**Figure 1:**
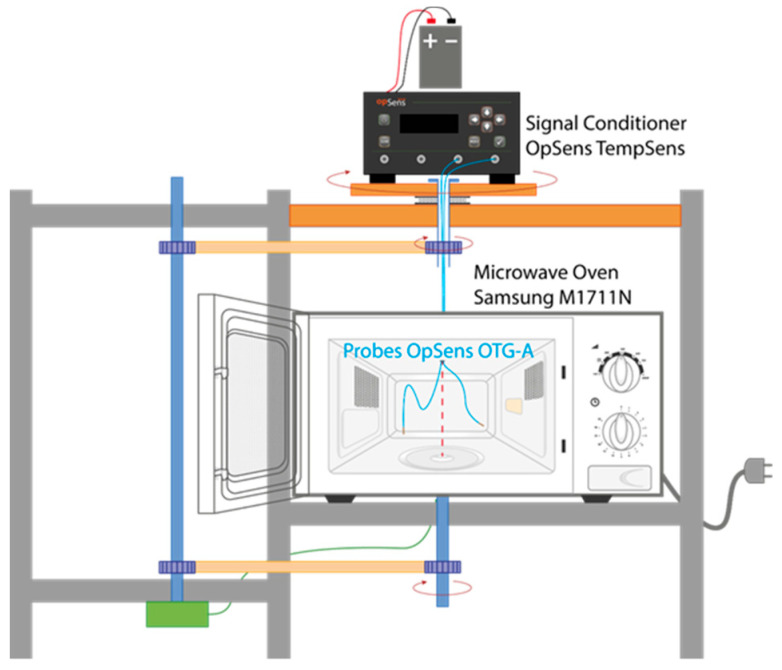
Scheme of the modified microwave oven for temperature-monitoring functionality inside the cavity.

An 800 W-power-rated Samsung M1711N microwave oven (Samsung Electronics, Bangkok, Thailand), with a cavity of 306 × 211 × 320 mm^3^, was used to irradiate batch samples at 2.45 GHz. This microwave oven was modified to monitor the temperature of the samples inside the cavity by means of GaAs temperature fiber optic sensors (OpSens OTG-A) and a signal conditioner (OpSens TempSens), whilst the microwave applicator operated normally (Figure 1), as described in Fayos et al. [17].

A prior offset temperature calibration was performed by comparing the readings of the optical temperature-measuring system and thermocouple-based temperature-measuring equipment used as a reference, whilst all sensors were stabilized at room temperature.

A set of five samples (80 mL each) per batch were processed in the microwave cavity, but only two samples per batch were temperature-monitored. The mass of the glass bottle and the tap was 146.2 g and 15.1 g, respectively. Both materials are transparent to microwave radiation and absorb heat by conduction and convection, as opposed to microwave absorption in beverages. The selection of processing parameters was mainly based on similar MW conditions required for ensuring microbial safety, which have previously been reported in other studies for plant-based beverages [15,16]. In this case, two power profiles were used to irradiate the samples. The 800-Watt-power-rated profile involves continuous MW irradiation at the maximum power that the MW oven can provide. The 600-Watt-power-rated profile is an on/off, pulsed MW irradiation providing the maximum power available with a duty cycle of 75%. It must be emphasized that this kind of general-purpose MW oven does not guarantee uniform irradiation at the rated power levels at any time. Nevertheless, these ovens are designed to provide acceptable time-averaged heating volumetric distributions in terms of temperature uniformity based on three main mechanisms: the excitation of multiple electrical patterns inside of the cavity (multimode irradiation), the rotation of the turntable, and the mixing produced by the convective flows within liquids, over a temperature gradient.

**Figure 2:**
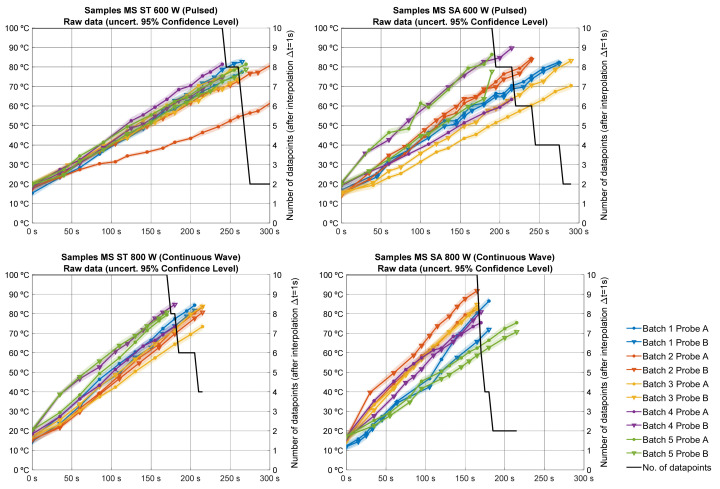
Temperature evolution dataset per specimen, power profile, batch, and probe. Also shown is the amount of datapoints per trace available after applying interpolation for the averaging process.

The MW-assisted pasteurization protocol was designed to process 5 batches per specimen (sucrose or stevia was added to beverages) at a specific power profile (800 W or 600 W), making a total of 20 tests, thus 40 temperature trace records (2 monitored samples per test), as depicted in Figure 2. 

**Figure 3:**
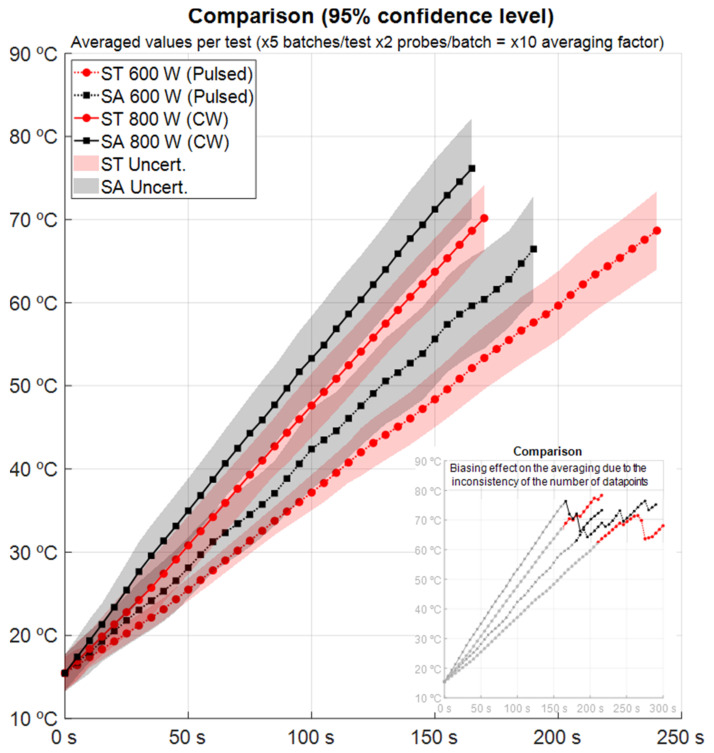
Temperature evolution per specimen and power profile (averaged values) and uncertainty associated (95% confidence level). Embedded subfigure shows the avoided biasing effect on the average that would have introduced the data dispersion when considering a non-constant number of datapoints (see Figure 2).

The averaging process among all the recorded data from different batches and probes required a prior interpolation of each trace with a harmonized uniform time sampling (Figure 3). To avoid biasing effects in the averaging process, the traces of each sensor were cropped to fit in the time span common to all sensor traces. As a result, each batch statistical curve was limited to the period of time where there were records from all the sensors of their respective batch test (10 averaged datapoints per unit of time and test). This processing technique is described in Fayos et al. [18].

**Figure 4:**
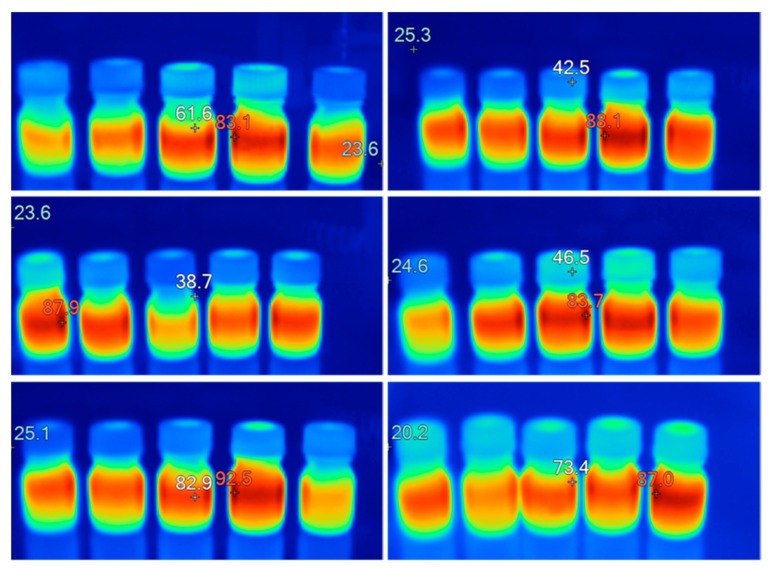
Thermographic imaging of some batches after being processed in the microwave. The locations in each thermography where the temperature levels are shown are indicated by the + symbol. The lowest temperature values are indicated by blue hues, while the intermediate and highest temperature levels are indicated by yellow and red hues, respectively.

Thermographic images of each processed batch were taken to evaluate the heating uniformity of the batch samples (Figure 4) by using a Fluke Ti25 IR Camera (Everett, Washington, DC, USA). These images show uneven heating among the samples of the same batch, but due to the convective flows, each sample showed an acceptable temperature homogeneity. This uneven behavior was detected by the monitoring probes, as they revealed different heating slopes (Figure 2).

#### 2.3.3. Conventional Pasteurization Processing

TP was conducted employing a Mastia thermoresistometer [19], applying a temperature range that starred from 20 °C. The thermoresistometer settings were established to implement a controlled heating ramp by 40 °C/min until reaching 85 °C, which was sustained for 15 s, according to common industrial standards applicable to beverages sharing similar compositional and pH features. Subsequently, the beverages underwent rapid cooling at a rate of 40 °C per minute, until 20 °C [7]. The processed beverages were stored in clear PET bottles as described below.

### 2.4. Sampling

Immediately after processing, all treated beverages were promptly transferred and stored in clear PET bottles (volume 30 mL; 80 mm × 27.5 mm Ø) with plastic screw caps and stored at 20 °C, protected from light, for 2 months. The beverages were designated with unique identification codes (Table 1). All experimental conditions were tested and analyzed in triplicate (*n* = 3), using the homogenized beverage, with the sample processed differently depending on the specific analysis considered. To determine the shelf life of the beverages, sampling was conducted on days 0, 7, 15, 30, 45, and 60. Additionally, untreated samples were preserved and analyzed to serve as reference controls in contrast to the treated ones on day 0.

### 2.5. Total Soluble Solids, Titratable Acidity, and pH

All parameters were measured as previously reported by Salar et al. [7].

### 2.6. Chromatographic Analysis of Phenolic Compounds

To set up the quantitative (poly)phenolic profile of the beverages, the methodology described by Salar et al. [6] was developed, using the identification and quantification criteria previously reported [6]. Anthocyanins were quantified as cyanidin 3-*O*-glucoside at 520 nm and flavanones as hesperidin at 280 nm. The concentration of phenolic compounds was expressed as mg/100 mL of sample.

### 2.7. Extraction and Quantification of Vitamin C

The vitamin C concentration was determined using the UHPLC-ESI-QqQ-MS/MS method described by Baenas et al. [20]. The quantification was achieved resorting to AA and DHAA standard curves prepared afresh on a daily basis during the analysis. The outcomes were recorded as mg/100 mL of juice.

### 2.8. Color Assessment

Color measurements were conducted using a Konica Minolta CM-5 Chroma Meter (Osaka, Japan). The results were expressed according to the CIEL*a*b* system, considering a light source calibrated to D65 and a visual angle of 10°, and including the analysis of Chroma (C*), hue angle (*h*), and total color differences (Δ*E*) [6].

### 2.9. Microbiological Analysis

The samples underwent aseptic dilution in buffered peptone water prior to analysis for aerobic mesophilic bacteria, molds and yeasts, aerobic psychrophilic bacteria, Enterobacteriaceae, and lactic acid bacteria (LAB) following the description provided in Salar et al. [7]. These assessments were conducted on day 0, immediately after processing, and then on days 7, 15, 30, 45, and 60 during storage at 20 °C. The objective of these microbial analyses was to evaluate the short- and long-term efficacy of MW heating pasteurization and TP throughout a 60-day shelf life. The results of microbial counts were reported as colony-forming units per milliliter (CFU/mL).

### 2.10. Statistical Analyses

The results were presented as the means ± SD (*n* = 3). A paired *t*-test was applied for the comparison of two parameters, while one-way analysis of variance (ANOVA) and Tukey’s multiple range test were conducted to compare three or more conditions. All statistical analyses were executed utilizing SPSS 19.0 software (LEAD Technologies, Inc., Chicago, IL, USA). The level of statistical significance was set at *p* < 0.05.

## 3. Results and Discussion

### 3.1. Initial Impact of Processing on the Measured Parameters 

#### 3.1.1. Influence on Physicochemical Parameters during Processing

The results for physicochemical parameters indicated a matching pH and titratable acidity for all beverages prior to any treatment, and remained stable immediately after processing, under the different treatments tested (Table 2). The TSS parameter was not significantly altered as a result of the treatments applied, with the only exception being the differences recorded between drinks prepared by the addition of sucrose and stevia as sweeteners, which allowed the identification of higher values when adding sucrose (Table 2). Nevertheless, interestingly, neither MW nor TP affected the pre-treated beverage values. These results suggested that no factor significantly affected the physicochemical properties of the beverages developed [21,22].

#### 3.1.2. Influence on Flavonoids

The anthocyanins found in the citrus–maqui beverages were from the maqui berry ingredient [6]. Their characteristics have been thoroughly described in previous studies conducted by Salar et al. [6,7], with delphinidin glycosides (3-*O*-sambubioside-5-*O*-glucoside, 3,5-*O*-diglucoside, and 3-*O*-sambubioside and 3-*O*-glucoside) and cyanidin glycosides (3-*O*-sambubioside-5-*O*-glucoside, 3,5-*O*-sambubioside, and 3-*O*-sambubioside, and 3-*O*-glucoside) being the main colored flavonoids. Regarding total anthocyanins, only slight statistically significant changes (*p* < 0.05) were observed after all the treatments, regardless of the added sweetener (Table 2). These outcomes suggested that the starting anthocyanin content was augmented to a similar extent after all the thermal treatments compared to the untreated beverage, with increases by 3% and 4%, on average, for sucrose and stevia, respectively, without significant differences between the treatments (*p* > 0.05). Similar results were found by Perez-Grijalva et al. [23]. In this sense, other authors have reported that the MW processing of plant-based foods leads to an increased rate of phenolic compound extraction [24,25]. This effect could potentially be linked to electroporation induced by electromagnetic waves, leading to cell wall rupture and the release of bioactive compounds into the juice, available to be absorbed in the gastrointestinal tract [21].

Concerning the flavanones, they were due to the citrus juices, with narirutin (naringenin 7-*O*-rutinoside), eriocitrin (eriodyctiol 7-*O*-rutinoside), and hesperidin (hesperetin 7-*O*-rutinoside) being the most abundant compounds. These flavanones were identified during preliminary studies conducted by Salar et al. [6,7]. Furthermore, regarding those beverages sweetened with sucrose, in terms of the total content of flavanones, there were no differences between the MW treatments, but these differences were significant (*p* < 0.05) when comparing them with TP. In this regard, TP induced an augment of flavanone concentrations of up to 30%. Thereby, this effect was more intense in comparison with both MW treatments, which were only able to increase the initial flavanone content by 10%, on average. On the other hand, concerning the stevia-sweetened beverages, significant differences (*p* < 0.05) were found across all processing treatments, rendering different percentages of increases (16%, 7%, and 19% for M1, M2, and TP, respectively).

This general growth in flavanones as a result of the thermal treatments could be linked to enhanced extraction efficiency because of the disturbance of the plant cell wall integrity, as previously reported in other studies on citrus juices [26]. Consequently, they render higher bioaccessibility when ingested.

#### 3.1.3. Influence on Vitamin C

Concerning vitamin C, a general decrease after the MW and TP treatments was observed for drinks sweetened by adding stevia or sucrose (*p* < 0.05). This loss was slightly higher for those beverages containing sucrose (14%, on average) than for those with added stevia (9%, on average) (Table 2). Regarding individual treatments, the results indicated that those beverages pasteurized using the highest irradiation power (800 W) presented a greater degradation of vitamin C (16% loss, on average) in comparison with those beverages obtained using stevia as a sweetener. Nevertheless, all conditions rendered vitamin C concentrations over 30% of the (EU) Nutrient Reference Value (80 mg/100 mL). These findings are consistent with other studies that have reported a significant reduction in ascorbic acid immediately after thermally processing plant-based foods [27,28].

#### 3.1.4. Influence on Color Parameters

It is worth mentioning that all treated samples, regardless of the sweetener or thermal processing used (SA/ST, MW/TP), exhibited significant differences in total color (Δ*E*) when compared with both (ST and SA) untreated beverages (*p* < 0.05) just after processing, as illustrated in Table 2. These changes are in agreement with previous authors [7,29]. However, it is noteworthy that these fluctuations in total color (Δ*E*) parameter, which did not exceed seven units in any of the treatment conditions, remained imperceptible to the naked eye in our beverages. This contrasts with other authors [30,31], who have reported that the human eye can discern the color alteration between samples with Δ*E* values exceeding four units in berry beverages.

#### 3.1.5. Influence on Microbiological Quality

Regarding the microbiological conditions, fresh samples presented an undetectable microbiological (molds and yeasts) concentration (<100 CFU/mL), including mesophilic aerobic bacteria, psychrophilic aerobic bacteria, Enterobacteriaceae, and lactic acid bacteria (<10 CFU/mL). In this regard, both MW and TP successfully maintained these values below the detection limits immediately after the application of treatments. Similar findings have previously been described by other authors [32,33]. In these works, microbial counts below the detection limits in plant-based beverages just after microwave processing or conventional pasteurization were described. Even though the untreated samples exhibited microbiological growth below the detection limits, thermal treatments were essential due to the aim of this study being to assess the beverages not only immediately after processing, but also over a real commercial shelf life—at least two months for this type of product. In this context, beverages that were not subjected to any thermal treatment underwent a complete deterioration of their microbiological profile in less than a week.

### 3.2. Impact of Storage on the Overall Measured Parameters

#### 3.2.1. Impact on Titratable Acidity, pH, and TSS during Storage

Regarding the findings related to pH and TA, there were no significant differences between sweeteners and the MW and TP treatments, as they were stable throughout the entire shelf-life period (60 days) (Table 3). Meanwhile, the percentages of variations in the total soluble solids expressed as °Brix were similar for both processing treatments (*p* > 0.05) (Table 3). However, there were notable divergences concerning both sweeteners, with higher values for beverages developed by adding sucrose.

These findings are in accordance with other authors who also reported no differences in the physicochemical properties during the monitored shelf-life period in similar drink matrices following MW or TP processing [6,7,16,34].

#### 3.2.2. Impact on Flavanones during Storage

**Figure 5:**
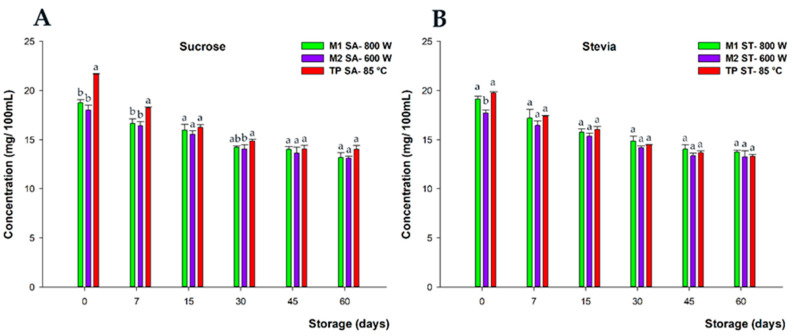
Total flavanone content (mg/100 mL) of beverages developed with sucrose (**A**) and stevia (**B**), subjected to microwave (M1 SA—800 W and M2—SA 600 W) and conventional pasteurization (TP SA—85 °C), measured during storage for 60 days and at 20 °C under darkness conditions. Bars with different lowercase letters within each time point were statistically different at *p* < 0.05, according to the analysis of variance (ANOVA) and Tukey’s multiple range test.

In relation to changes occurring during the storage period concerning the total flavanone content, statistical significant differences (*p* < 0.05) were observed between both MW and TP treatments in the frame of the first week of storage for sucrose-sweetened drinks (Figure 5).

These differences should not be taken into consideration from a practical perspective when the total amount of flavanones and their possible biological power is considered, as other factors (e.g., such as citrus varieties and agroclimatic conditions, among others), would give rise to even more significant initial differences between batches of untreated juices. On the other hand, no significant differences (*p* > 0.05) were found between treatments throughout the entire preservation period for stevia-sweetened beverages. In this context, it is noteworthy that even if some slight differences in losses could be observed during storage, after the second week until the 60-day shelf-life period, all the treatments presented the same flavanone concentration. This protective effect of microwave treatments was also reported by Zhia et al. [35]; meanwhile, the preserving role of stevia on flavanones during storage, after conventional pasteurization, has previously been described by Salar et al. [6].

Considering the relative abundance of individual flavanones, neither narirutin nor eriocitrin nor *O*-glycosyl-naringenin showed a significant decrease during storage. Beyond this, hesperidin was the most affected flavanone, accordingly with previous works [6,7]. Therefore, the final losses in terms of total flavanone content were primarily due to the degradation of hesperidin under all processing conditions throughout the beverage’s shelf life, as previously reported [36].

#### 3.2.3. Impact on Anthocyanins during Storage

**Figure 6:**
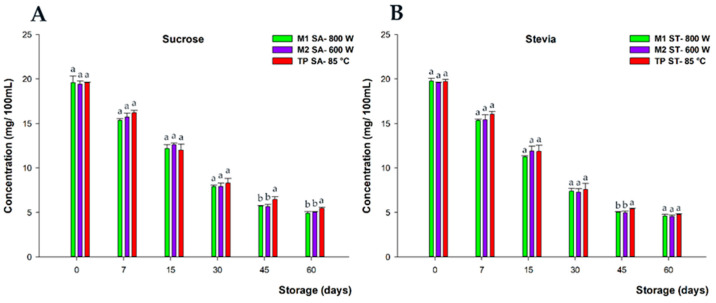
Total anthocyanin content (mg/100 mL) of beverages developed with sucrose (**A**) and stevia (**B**), subjected to microwave (M1 SA—800 W and M2—SA 600 W) and conventional pasteurization (TP SA—85 °C), measured during storage for 60 days and at 20 °C under darkness conditions. Bars with different lowercase letters within each time point were statistically different at *p* < 0.05, according to the analysis of variance (ANOVA) and Tukey’s multiple range test.

The concentration of total anthocyanins at day 0 of the shelf life was similar across the processing treatments and for both sweeteners (19.60 mg/100 mL, on average for both sweeteners). Regarding the degradation rate of anthocyanins during the shelf-life period, the highest percentage of loss occurred during 45 days of storage, with no significant differences between MW and TP treatments (*p* > 0.05), reaching percentages by 59% and 62% loss, on average, across the three processing treatments, for sucrose and stevia, respectively (Figure 6). Finally, the degradation rate of anthocyanins slowed down between days 45 and 60, with slightly higher final loss percentages for stevia compared to sucrose, with cumulative losses of 77% and 74%, on average, respectively, at the end of the shelf-life period at 20 °C.

In this frame, the preservative influence of sucrose on anthocyanins has been described previously [37,38]. Furthermore, the kinetics of anthocyanin degradation during storage is in agreement with previous descriptions in the literature [6]. Moreover, other work comparing HHP and TP [7] found and discussed similar results, indicating that MW could also be a valuable alternative to the non-thermal process (HHP).

Furthermore, the degradation of anthocyanins in the frame of the present work, and given the experimental conditions described above, could largely be attributed to the adverse influence of vitamin C on the stability of these colored polyphenols [39,40]. This is mainly due to two mechanisms of mutual degradation, which have been described in previous studies by our group [6,7].

#### 3.2.4. Impact of MW and TP on the Stability of Vitamin C during Storage

**Figure 7:**
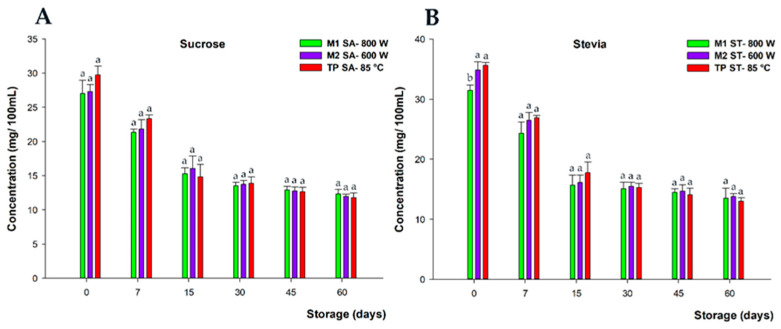
Vitamin C content (mg/100 mL) of beverages developed with sucrose (**A**) and stevia (**B**), subjected to microwave (M1 SA—800 W and M2—SA 600 W) and conventional pasteurization (TP SA—85 °C), measured during storage for 60 days and at 20 °C under darkness conditions. Bars with different lowercase letters within each time point were statistically different at *p* < 0.05, according to the analysis of variance (ANOVA) and Tukey’s multiple range test.

The initial vitamin C content in all processed beverages did not exhibit significant differences among the treatments with each sweetener, averaging 34 mg/100 mL and 28 mg/100 mL for stevia and sucrose, respectively. Overall, during the 60-day shelf-life monitoring, the results demonstrated that during the initial fortnight, vitamin C underwent a rapid and significant decrease (*p* < 0.05) (Figure 7), with average losses of 45% and 57% across the three thermal treatments for sucrose- and stevia-sweetened beverages, correspondingly. However, after day 15, this loss rate slowed down, and degradation was less pronounced until the end of storage, resulting in final losses of 59% on average for both sweeteners. Similar percentages of vitamin C loss have been reported in other preservation studies [6,41]. 

This significant degradation of vitamin C during storage at room temperature might be due, not only to the mutual degradation between vitamin C and anthocyanins, as previously mentioned, but also to the storage temperature, which has been extensively described by other authors as the decisive factor in the preservation of this bioactive compound in food matrices during shelf life [42].

Concerning the individual treatments, no significant differences (*p* > 0.05) were observed between the MW-treated beverages and those treated using conventional pasteurization, for both stevia and sucrose. Similar findings were described by Géczi et al. [43]. Furthermore, all three thermal processing treatments managed to preserve a significant percentage of the vitamin C content up to the 60-day shelf-life period. In this sense, contrasting evidence on this issue has been previously reported regarding vegetable- and fruit-based products. Igual et al. [13] and Benlloc-Tinoco et al. [44] described a higher vitamin C content in MW-treated samples compared with TP ones during storage. On the other hand, these results also indicated that the MW treatment could be better that HHP, previously compared to conventional pasteurization [7].

#### 3.2.5. Color Changes of Beverages during Storage

The CIEL*a*b* color parameters and their evolution were monitored over 60-day storage at 20 °C. The reddish hue of the citrus–maqui beverage was attributed to the overall concentration of colored polyphenols, which contributes to an appealing sensory tonality for consumers.

Regarding the evolution of the different parameters determining color, the lightness value (CIEL*) tended to increase over the course of the 60-day storage for the three thermal treatments and with both sweeteners, resulting in similar final values under all conditions (Table 4 and Table 5). This degradation in luminosity was attributed to the breakdown of anthocyanins during shelf life [45].

On the other hand, a general trend towards a decrease in the CIEa* value was found during storage for all beverages, regardless of the technological treatments or sweeteners used (Table 4 and Table 5). However, for beverages sweetened with stevia, this decrease was slightly more pronounced relative to the beverages sweetened with sucrose, which was consistent with the rate of degradation of the total anthocyanin content in the beverages, as previously discussed. Additionally, it has to be stressed that the attractive reddish coloration was stably preserved over the 2 months of storage under all conditions. Furthermore, these subtle variations in the reddish coloration of the beverages throughout the shelf life were attributed to colored polymers formed through polymerization reactions between anthocyanins and other phenolic compounds (flavones, ferulic acid, flavanols, etc.), which dull any negligible color or changes [46,47].

Regarding the evolution during storage in the yellowness (CIEb*) value, it was characterized by small increments during shelf life, observed consistently across all processing treatments and with both sweeteners (Table 4 and Table 5). On the other hand, while the Chroma parameter (C*) exhibited a very slight decrease for all conditions during storage, indicating a subtle loss of brightness in the beverages by the end of the storage period. Conversely, the hue angle (*h*) parameter displayed a general increase in all drinks over their shelf life. This suggests a numerical tendency toward browning, although it was hardly discernible to the naked eye.

Finally, the total color difference, measured as Δ*E*, increased significantly (*p* < 0.05) over the 60-day storage of the beverages, in all the evaluated conditions (Table 4 and Table 5), indicating significant color variations (*p* < 0.05) among different shelf-life periods for the citrus–maqui beverages. It is noteworthy that these color differences have been visually discernible to the human eye at values of 3.0 to 4.0 units for Δ*E* [48,49]. Nevertheless, in our study, these visual differences were not detectable to the naked eye until the Δ*E* value exceeded 14 units after one month of storage, maintaining a stable reddish coloration for all beverages, regardless of experimental conditions.

In summary, the use of both MW and TP treatments significantly impacted the color of the drinks during their shelf life, although with minimal differences between treatments. On the contrary, Marszałek et al. [41] pointed out notable differences between MW processing and traditional thermal treatments. On the other hand, there were only slight variations in color when comparing the use of stevia and sucrose as sweeteners, as both of them induced color alterations in a similar way.

#### 3.2.6. Alterations in Microbial Characteristics throughout Storage

The microbial characteristics of the citrus–maqui beverage samples subjected to MW (MW-800 W and MW-600 W) and TP (TP-85 °C) were assessed over 60 days of storage at 20 °C, for citrus and maqui-based beverages with added stevia and sucrose. In the present study, microbial quality was not altered in any of the processed drink formulations throughout the 60-day storage period, as evidenced by the maintenance of the microbial burden below the limits of quantification of the analytical technique applied for all the microorganisms monitored. These findings indicated that the blends of citrus and maqui were microbiologically safe and remained stable throughout their shelf life. The inactivation capacity of the MW treatments is based on its thermal increase. These outcomes agreed with the information available in the literature, which also reported the efficacy of both MW and TP in ensuring microbiological safety in other vegetable- and fruit-based juices [23,35]. This potential antimicrobial effect of the application of the MW technology might be attributed to four main theories: selective heating, magnetic field coupling, cell membrane rupture, and electroporation [9,34,50].

## 4. Conclusions

Considering these results, MW treatment is a promising method for obtaining beverages with a high content of health-related compounds that could be an efficacious alternative to conventional thermal (TP) or non-thermal treatments (such as HHP, among others) within the beverage industry. In terms of energy, low-energy pasteurization (600 W) rendered the same results as high energy, indicating that 600 W would be sufficient, yielding subsequent energy savings.

After revealing the application of the obtained results, future trends in the research on sweetener and thermal treatment combinations (gaps of knowledge) should be studied, for different beverages, as general conditions cannot be applied to all.

## Figures and Tables

**Table 1:** Encoding of samples.

Code	Beverage and Storage Conditions
Control	Untreated sample
M1 SA—800 W	Beverage with added sucrose subjected to microwave pasteurization (800 W) stored at 20 °C
M2 SA—600 W	Beverage with added sucrose subjected to microwave pasteurization (600 W) stored at 20 °C
M1 ST—800 W	Beverage with added stevia subjected to microwave pasteurization (800 W) stored at 20 °C
M2 ST—600 W	Beverage with added stevia subjected to microwave pasteurization (600 W) stored at 20 °C
TP SA—85 °C	Beverage with added sucrose subjected to conventional pasteurization stored at 20 °C
TP ST—85 °C	Beverage with added stevia subjected to conventional pasteurization stored at 20 °C

**Table 2:** Physicochemical characteristics and antioxidant biomolecules of non-processed, microwaved, and pasteurized citrus–maqui beverages at zero time of storage.

Condition ^Z^	Physicochemical Parameters			Bioactive Compounds (mg/100 mL)			Color (ΔE)
	pH	TA (g CA/100 mL)	TSS (°Brix)	Anthocyanins	Flavanones	Vitamin C	
Non-processed-SA	3.39 a ^Y^	3.44 ab	15.80 b	18.85 b	16.72 c	32.46 a	0.00 d
M1 SA—800 W	3.38 ab	3.47 a	16.00 a	19.60 a	18.75 b	27.00 b	5.16 a
M2 SA—600 W	3.35 c	3.42 b	15.80 b	19.41 a	17.99 b	27.32 b	1.63 c
TP SA—85 °C	3.36 bc	3.41 b	15.80 b	19.57 a	21.65 a	29.72 ab	4.43 b
LSD (*p* < 0.05)	<0.01	0.01	<0.01	<0.01	<0.01	0.01	<0.01
*p*-value	*** ^X^	**	***	***	***	**	***
Non-processed-ST	3.34	3.72	9.00 b	18.91 b	16.53 d	37.27 a	0.00 d
M1 ST—800 W	3.36	3.69	9.20 a	19.76 a	19.14 b	31.49 c	6.33 a
M2 ST—600 W	3.35	3.74	9.00 b	19.55 a	17.70 c	34.87 b	4.68 c
TP ST—85 °C	3.34	3.68	9.20 a	19.70 a	19.73 a	35.65 ab	5.32 b
LSD (*p* < 0.05)	0.09	0.15	0.01	<0.01	<0.01	<0.01	<0.01
*p*-value	N.s.	N.s.	**	***	***	***	***

^Z^ Non-processed-SA, fresh unprocessed beverage with sucrose added; M1 SA—800 W, microwave at 800 W/sucrose; M2 SA—600 W, microwave at 600 W/sucrose; TP SA—85 °C, conventional pasteurization at 85 °C/sucrose; Non-processed-ST, fresh unprocessed beverage with stevia added; M1 ST—800 W, microwave at 800 W/stevia; M2 ST—600 W, microwave at 600 W/stevia; TP ST—85 °C, conventional pasteurization at 85 °C/stevia. ^Y^ Values within each column followed by different lowercase letters are significantly different at *p* < 0.05 according to the ANOVA and Tukey’s multiple range test. ^X^ Significant at ** (*p* < 0.01), and *** (*p* < 0.001). N.s., not significant.

**Table 3:** pH, titratable acidity (TA), and total soluble solids (TSS) measured at day 0 (initial) and after 60 days of storage (final) for beverages subjected to MW and TP and stored at 20 °C.

Condition ^Z^	pH			TA (g CA/100 mL)			TSS (°Brix)		
	Initial	Final	*p*-Value	Initial	Final	*p*-Value	Initial	Final	*p*-Value
M1 SA—800 W	3.38 a ^Y^	3.46 a	*** ^X^	3.47 a	3.48	N.s.	16.00 a	16.00 a	N.s.
M2 SA—600 W	3.35 b	3.44 b	***	3.42 b	3.46	N.s.	15.80 b	15.80 b	N.s.
TP SA—85 °C	3.36 b	3.45 ab	***	3.41 b	3.49	**	15.80 b	15.80 b	N.s.
LSD (*p* < 0.05)	0.01	0.02		0.01	0.56		0.00	0.00	
*p*-value	**	*		**	N.s.		***	***	
M1 ST—800 W	3.36	3.44	***	3.69	3.75	N.s.	9.20 a	9.20	N.s.
M2 ST—600 W	3.35	3.44	***	3.74	3.78	N.s.	9.00 b	9.20	*
TP ST—85 °C	3.34	3.44	***	3.68	3.74	N.s.	9.20 a	9.20	N.s.
LSD (*p* < 0.05)	0.10	0.79		0.16	0.43		0.00	1.00	
*p*-value	N.s.	N.s.		N.s.	N.s.		***	N.s.	

^Z^ M1 SA—800 W, microwave at 800 W/sucrose; M2 SA—600 W, microwave at 600 W/sucrose; TP SA—85 °C, conventional pasteurization at 85 °C/sucrose; M1 ST—800 W, microwave at 800 W/stevia; M2 ST—600 W, microwave at 600 W/stevia; TP ST—85 °C, conventional pasteurization at 85 °C/stevia. ^Y^ Data (mean) within each column, values followed by different lowercase letters for each processing condition are significantly different at *p* < 0.05 according to the analysis of variance (ANOVA) and Tukey’s multiple range test. ^X^ Significant at * (*p* < 0.05), ** (*p* < 0.01), and *** (*p* < 0.001) according to a paired *t*-test. N.s., not significant.

**Table 4:** Stability of CIEL*a*b* values in beverages with sucrose added and stored at 20 °C.

Parameter	Storage (Days)	M1 SA—800 W	M2 SA—600 W	TP SA—85 °C	LSD (*p* < 0.001)
CIEL*	0	31.08 aA ^Z^	33.43 aB	31.31 aA	0.88
	7	32.77 aA	33.72 aA	32.38 bA	1.64
	15	36.71 bA	39.12 bB	36.73 cA	1.22
	30	40.02 cA	40.42 bA	39.07 dA	1.15
	45	42.99 dB	42.45 cB	40.20 eA	1.38
	60	41.01 cdA	43.68 cB	40.81 eA	0.68
	LSD (*p* < 0.001)	1.29	1.18	0.61	
CIEa*	0	57.96 eA	59.33 eB	58.09 eA	0.63
	7	57.00 deA	56.89 dA	56.77 dA	0.98
	15	56.02 dA	56.18 dA	56.20 dA	0.98
	30	51.18 cA	51.36 cA	51.33 cA	0.36
	45	48.09 bB	46.79 bA	47.82 bAB	0.83
	60	44.55 aA	45.30 aA	45.71 aA	1.23
	LSD (*p* < 0.001)	0.67	0.82	0.85	
CIEb*	0	37.18 aB	34.73 aA	36.44 aB	0.63
	7	36.20 aA	35.98 aA	36.17 aA	0.98
	15	36.90 aB	34.98 aA	35.49 aAB	1.28
	30	41.07 bA	40.99 bA	40.55 bA	0.72
	45	42.37 bA	45.64 cB	44.20 cAB	1.46
	60	47.35 cB	44.46 cA	43.91 cA	1.87
	LSD (*p* < 0.001)	1.21	1.33	0.76	
Chroma (C*)	0	68.87 dB	68.75 dAB	68.58 eA	0.79
	7	67.53 cA	67.32 cA	67.31 dA	0.48
	15	67.08 cB	66.18 bA	66.47 cAB	0.55
	30	65.62 bA	65.71 bA	65.41 bA	0.32
	45	64.09 aA	65.37 bB	65.11 bAB	0.97
	60	65.01 abB	63.48 aA	63.39 aA	0.83
	LSD (*p* < 0.001)	0.687	0.63	0.20	
Hue angle (*h*)	0	32.68 aB	30.34 aA	32.10 aB	0.17
	7	32.42 aA	32.32 bA	32.51 aA	1.18
	15	33.37 aA	31.90 abA	32.27 aA	1.30
	30	38.74 bB	38.60 cA	38.31 bA	0.64
	45	41.38 cA	44.28 dB	42.74 cA	1.21
	60	46.77 dB	44.46 dAB	43.85 cA	1.86
	LSD (*p* < 0.001)	1.04	1.25	0.99	
Δ*E*	0	0.00 a	0.00 a	0.00 a	<0.01
	7	2.18 bAB	3.08 bB	1.73 bA	0.99
	15	6.03 cA	6.51 cA	5.84 cA	1.24
	30	11.91 dAB	12.32 dB	11.09 dA	0.90
	45	16.36 eA	18.92 eB	15.65 eA	1.49
	60	19.58 fB	19.94 eB	17.33 fA	1.56
	LSD (*p* < 0.001)	0.94	1.25	0.86	

^Z^ Means (*n* = 3) within a column followed by different lowercase letters (storage time point comparison) or different capital letters within a row (treatment comparison) are significantly different at *p* < 0.001.

**Table 5:** Stability of CIEL*a*b* values in beverages with stevia added and stored at 20 °C.

Parameter	Storage (Days)	M1 ST—800 W	M2 ST—600 W	TP ST—85 °C	LSD (*p* < 0.001)
CIEL*	0	31.30 aA ^Z^	32.43 aC	31.52 aB	0.09
	7	34.59 bB	32.75 aA	33.26 bA	0.82
	15	37.78 cB	38.15 bB	36.20 cA	0.54
	30	41.94 dC	40.45 cB	38.55 dA	0.65
	45	42.99 eB	44.30 dB	39.62 eA	1.15
	60	42.68 eA	43.05 dA	42.44 fA	0.76
	LSD (*p* < 0.001)	0.37	0.96	0.51	
CIEa*	0	58.01 eA	58.68 eC	58.31 fB	0.17
	7	57.84 eB	56.16 dA	57.21 eA	0.69
	15	55.67 dA	56.02 dA	55.71 dA	0.32
	30	49.28 cA	52.34 cB	50.57 cA	1.23
	45	45.97 bA	46.00 bA	46.28 bA	0.45
	60	42.79 aB	43.70 aC	42.28 aA	0.28
	LSD (*p* < 0.001)	0.35	0.71	0.57	
CIEb*	0	37.69 bB	36.72 aA	36.36 aA	0.16
	7	36.50 aA	37.75 aB	36.51 aA	0.51
	15	37.38 abA	37.07 aA	37.04 aA	0.61
	30	42.97 cB	40.25 bA	41.57 bAB	1.20
	45	46.12 dA	45.11 cA	44.96 cA	1.12
	60	48.95 eB	47.73 dA	47.80 dA	0.67
	LSD (*p* < 0.001)	0.65	0.81	0.66	
Chroma (C*)	0	69.18 dB	69.17 dB	68.87 fA	0.14
	7	68.39 cB	67.67 cA	67.87 eA	0.33
	15	67.06 bA	67.19 cA	66.90 dA	0.27
	30	65.38 aA	66.04 bB	65.47 cA	0.28
	45	65.13 aA	64.43 aA	64.53 bA	0.68
	60	65.01 abB	64.72 aB	63.81 aA	0.35
	LSD (*p* < 0.001)	0.28	0.48	0.16	
Hue angle (*h*)	0	33.01 abC	31.97 aA	32.16 aB	0.16
	7	32.26 aA	33.91 bB	32.54 abA	0.67
	15	33.88 bA	33.49 bA	33.61 bA	0.55
	30	41.08 cB	37.56 cA	39.42 cB	1.48
	45	45.09 dA	44.44 dA	44.16 dA	0.89
	60	48.84 eB	47.51 eAB	48.51 eB	0.56
	LSD (*p* < 0.001)	0.59	0.83	0.75	
Δ*E*	0	0.00 a	0.00 a	0.00 a	<0.01
	7	3.51 bB	2.84 bAB	2.05 bA	0.74
	15	6.91 cC	6.33 cB	5.35 cA	0.46
	30	14.75 dAB	10.84 dA	11.54 dA	1.56
	45	18.80 eB	19.34 eC	16.69 eA	0.42
	60	22.15 fAB	21.45 fA	22.35 fB	0.57
	LSD (*p* < 0.001)	0.42	0.95	0.63	

^Z^ Means (*n* = 3) within a column followed by different lowercase letters (storage time point comparison) or different capital letters within a row (treatment comparison) are significantly different at *p* < 0.001.

## Data Availability

Data is contained within the article.
